# Diels-Alder reactions using 4,7-dioxygenated indanones as dienophiles for regioselective construction of oxygenated 2,3-dihydrobenz[*f*]indenone skeleton

**DOI:** 10.3762/bjoc.4.15

**Published:** 2008-05-15

**Authors:** Natsuno Etomi, Takuya Kumamoto, Waka Nakanishi, Tsutomu Ishikawa

**Affiliations:** 1 Graduate School of Pharmaceutical Sciences, Chiba University, 1-33 Yayoi-cho Inage-ku, Chiba 263-8522 Japan. Fax: +81-43-290-2911

## Abstract

Regioselective construction of 4,8,9-trioxygenated 2,3-dihydrobenz[*f*]indenones, key intermediates for the synthesis of kinamycin antibiotics, was achieved via Diels-Alder reactions (DAR) using 4,7-dioxygenated indanone-type compounds as dienophiles. Reaction of indanetrione with 1-methoxybutadiene gave a 1 : 1 mixture of undesired 4,5,9-trioxygenated 2,3-dihydrobenz[*f*]indenone and [4.4.3]propellane. The addition of Lewis acid did not affect the product ratio, whereas the use of the 6-bromoindanetrione exclusively afforded the latter propellane. On the other hand, DAR of benzyne derived from bromoindan and furan gave 5,8-epoxy-2,3-dihydrobenz[*f*]indene, which was subjected to acid-induced ring opening to give 2,3-dihydrobenz[*f*]indenone with undesired 4,5,9-trioxy functions.

## Background

**Figure 1:**
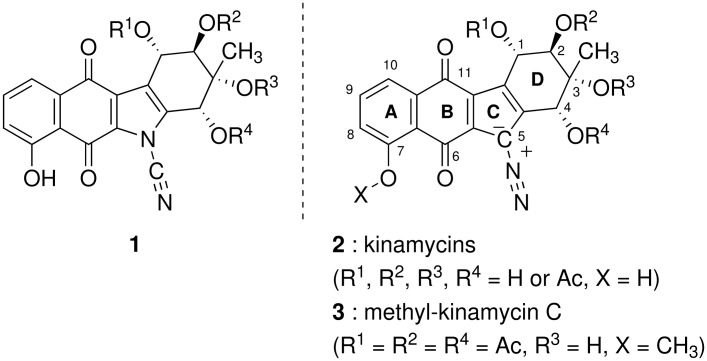
The structure of kinamycins.

**Scheme 1:**
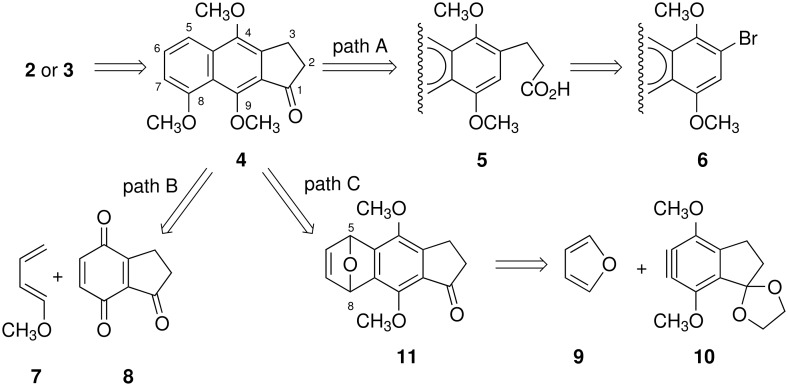
Retrosynthesis of kinamycins.

Kinamycins, isolated from *Streptomyces murayamaensis* sp. nov. Hata et Ohtani in 1970 [1–3], have attracted attention due to their antibiotic and antitumor activities [7–10]. These compounds had been originally characterized as cyanamides **1** with a linearly-fused 6-6-5-6 membered ring system [11–12]; however, the structure was revised to diazoalkanes **2** by spectroscopic means [13–14] and by total synthesis [15–17] (Figure 1). In our total synthesis of methyl-kinamycin C (**3**) [21], regioselective synthesis of 4,8,9-trioxygenated 2,3-dihydrobenz[*f*]indenone **4** [22] was a key issue, which was achieved via C ring construction with intramolecular Friedel-Crafts reaction of naphthalenepropanoic acid **5** (path A, Scheme 1) [23]. However, the utilization of a stoichiometric amount of expensive silver salt for the synthesis of bromonaphthalene **6** [24] hampered large-scale synthesis of **4**. Towards a solution to this problem, we planned the synthesis of **4** via A-ring construction by DAR of indanone-type compounds and oxygenated dienes: *i. e.* 1) DAR of indanetrione **8** and 1-methoxy-1,3-butadiene (**7**) (path B; quinone route) [28]; 2) regioselective ring-opening of 5,8-epoxy-2,3-dihydrobenz[*f*]indenone **11** derived from benzyne **10** and furan (**9**) (path C, benzyne route). Now we report that both of the methods are effective for the construction of the 2,3-dihydrobenz[*f*]indenone skeleton, but not for the regioselective synthesis of the desired 4,8,9-trioxygenated ones.

## Results and Discussion

**Scheme 2:**
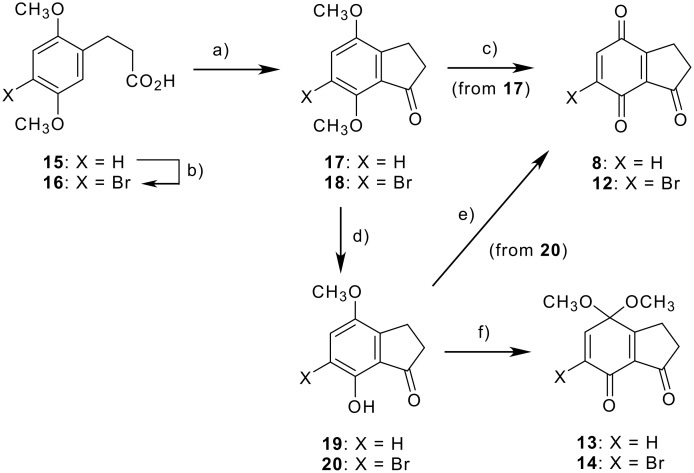
Synthesis of quinones **8** and **12** and the acetals **13** and **14**. *Reagents and conditions:* a) P_2_O_5_, CH_3_SO_3_H, CH_2_Cl_2_ or CHCl_3_ (67% for **17**, 71% for **18**); b) bromine, 1,4-dioxane, H_2_O, rt, 2 h (68%); c) CAN, CH_3_CN, H_2_O, 0 °C, 30 min (65%); d) MgI_2_ · 6H_2_O, benzene, Dean-Stark (81% for **19**, 96% for **20**); e) PIFA, H_2_O, CH_3_CN, rt, 30 min (86%); f) PIFA, CH_3_OH, CH_3_CN, 0 °C (74% for **13**, 92% for **14**).

In the quinone route, we designed several indanetriones to modulate steric and electronic factors; *i.e.* 1,4,7-indanetrione **8**, the 6-brominated quinone **12**, and the corresponding 4-monoacetals **13** and **14** (Scheme 2). Intramolecular Friedel-Crafts reaction of 2,5-dimethoxybenzenepropanoic acid (**15**) and the 4-brominated derivative **16** [31], by a procedure modified from the synthesis of **4** [23], afforded the corresponding indanones **17** and **18**. Cerium ammonium nitrate (CAN) oxidation [32] of indanone **17** smoothly afforded indanetrione **8**, but attempts with bromoindanone **18** resulted in no reaction even under reflux. Utilization of a milder oxidant phenyliodosyl bis(trifluoroacetate) (PIFA) [33] for the oxidation of phenolic indanone **20**, derived from **18** by selective demethylation with magnesium iodide (MgI_2_) [34], gave bromoquinone **12** after modification of the workup protocol without aqueous sodium bicarbonate. The PIFA oxidation of phenols **19** [35] and **20** in the presence of methanol gave the corresponding monoacetals **13** and **14**.

**Figure 2:**
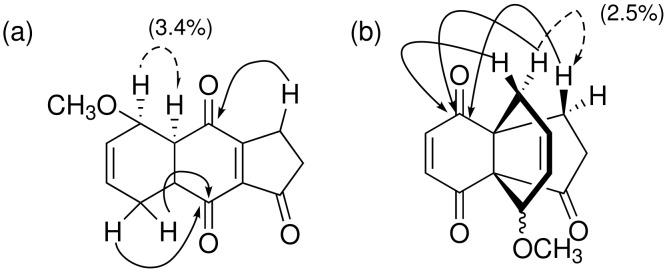
Selected HMBC correlations (lines) and NOE enhancements (dash) on **21** (a) and on **22** (b).

DAR of indanetrione **8** and 1-methoxy-1,3-butadiene (**7**) in dichloromethane (CH_2_Cl_2_) proceeded smoothly at −16 °C to give a 1 : 1 mixture of 2,3-dihydrobenz[*f*]indenone **21** and [4.4.3]propellane **22**, produced by participation of the double bond at the ring junction in **8** (entry 1 in Table 1). Both compounds were obtained as single diastereoisomers. The former was determined as an undesired 5-methoxy derivative **21**, the structure of which was deduced by HMBC correlations and NOE enhancement (Figure 2a). The latter structure **22** was also determined by HMBC and NOE experiments (Figure 2b); however, the relative configuration of the carbon connected to the methoxy group could not be determined because of lacking NOE data. Next, the effect of Lewis acid on the regioselectivity was examined. At first, zinc chloride (ZnCl_2_), an effective catalyst on DAR of benz[*f*]indenone and Danishefsky-type diene [21], was chosen. Addition of a catalytic amount of ZnCl_2_ at −78 °C did not affect the regioselectivity (entry 2). An increase in the amount of ZnCl_2_ led to the formation of a complex mixture containing a small amount of propellane **22** (entry 3). Next, instead of ZnCl_2_, which is only slightly soluble in CH_2_Cl_2_, boron trifluoride etherate (BF_3_ · OEt_2_) was applied as a soluble Lewis acid; however, similar results were obtained to those with ZnCl_2_ (entries 4, 5). Interestingly, when bromoquinone **12** was reacted under the conditions of entry 1, DAR at the ring junction proceeded exclusively to give bromopropellane **23** in high yield as the sole product (entry 6). The yield was slightly reduced and the regioselectivity was not affected in the reaction in the presence of a catalytic amount of BF_3_ · OEt_2_ (entry 7).

We next turned to the use of quinone monoacetals **13** and **14** as dienophiles [36]. No adduct was formed on reaction of **13** in CH_2_Cl_2_ without a catalyst at room temperature (rt) (entry 8), whereas refluxing in toluene gave deprotected propellane **22** (16%) together with phenol **19** (23%), a synthetic precursor of **13** (entry 9). Stirring bromoquinone monoacetal **14** in CH_2_Cl_2_ at rt yielded only a small amount of propellane **23** (entry 10). The regioselectivity and the yield were not improved by the addition of ZnCl_2_, from which the phenol **20** and propellane **23** were isolated in low yields as conversion products (entry 11). The desired 4,8,9-trioxygenated 2,3-dihydrobenz[*f*]indenone-type compound **24** was not obtained in any of the experiments.

**Table 1:** DARs of dienophiles **8**, **12-14** and diene **7.**

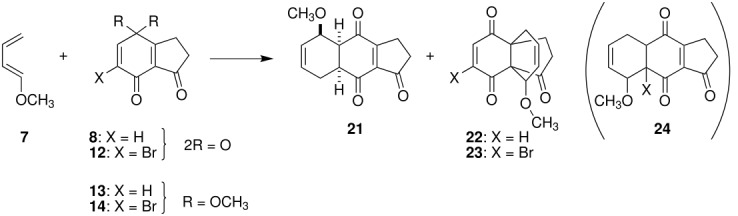					
Entry	Dienophile	Solvent	Additive	Conditions	Results

1	**8**	CH_2_Cl_2_	-	−16 °C, 4 h	**21** (47%), **22** (42%)*^a^*
2	**8**	CH_2_Cl_2_	ZnCl_2_ (0.14 eq)	−78 °C, 4 h	**21** : **22** = *ca.* 1 : 1^b^
3	**8**	CH_2_Cl_2_	ZnCl_2_ (1 eq)	−78 °C, 2 h	**22** (9%)^a^
4	**8**	CH_2_Cl_2_	BF_3_ · OEt_2_ (0.14 eq)	−78 °C, 2 h	**21** : **22** = *ca.* 0.8 : 1^b, c^
5	**8**	CH_2_Cl_2_	BF_3_ · OEt_2_ (1 eq)	−78 °C, 2 h	CM^d^
6	**12**	CH_2_Cl_2_	-	−16 °C, 4 h	**23** (89%)^a^
7	**12**	CH_2_Cl_2_	BF_3_ · OEt_2_ (0.14 eq)	−78 °C, 2 h	**23** (57%)^a^
8	**13**	CH_2_Cl_2_	-	rt, 8 h	NR^e^
9^f^	**13**	toluene	-	120 °C, 9 h	**19** (21%), **22** (16%)^a^
10	**14**	CH_2_Cl_2_	-	rt, 6 h	**23** (4%)^a^
11	**14**	CH_2_Cl_2_	ZnCl_2_ (0.20 eq)	rt, 2 h	**20** (15%), **23** (8%)^a^

^a^Isolated yield(s). ^b^Estimated by ^1^H NMR of crude product. ^c^**21** (4%) and **22** (11%) were isolated after column chromatography. ^d^A complex mixture. ^e^No reaction. ^f^Performed in a sealed tube

**Scheme 3:**
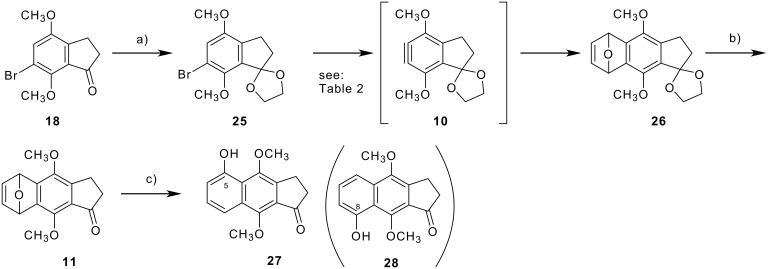
DAR of benzyne **10** and furan (**9**). *Reagents and conditions: *a) ethylene glycol, PPTS, benzene, reflux, 17 h (89%), b) *conc*. HCl, methanol, reflux, 19 h (50%); c) *conc*. HCl, methanol, THF, reflux, 38 h (39%, with recovery of **11** in 42%).

**Figure 3:**
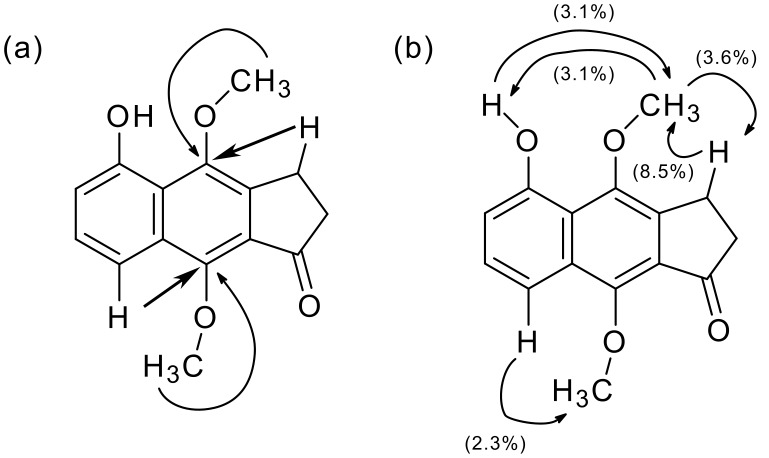
Selected HMBC correlations (a) and NOE enhancements (b) on the ring-opened product **27**.

Next, synthesis of 2,3-dihydrobenz[*f*]indenone via a benzyne route was examined by treatment of bromoindanone acetal **25**, prepared from bromoindanone **18**, with a base in the presence of furan (**9**) (Scheme 3). Application of Giles' protocol [41] using sodium amide as a base in the presence of a large excess amount (*ca.* 15 equivalents) of furan (**9**) in THF gave 5,8-epoxy-2,3-dihydrobenz[*f*]indene **26** in 3% yield together with recovery of the starting **25** (79%) (entry 1 in Table 2). The yield was still low (12%) under microwave irradiation (entry 2). The use of lithium diisopropylamide (LDA) [42] in THF slightly increased the yield of **26** to 25% (entry 3). Although no improvement was observed after increasing the quantity of the base (entry 4), the yield was slightly improved to 30% on decreasing the quantity of furan (**9**) to two equivalents (entry 5). Bases derived from tetramethylpiperidine (TMP) [43] were not effective (entries 6, 7). Thus, the desired improvement of the yield was not observed for the synthesis of the 5,8-epoxybenz[*f*]indene derivative **26**; nevertheless, the ring-opening step was examined. Treatment of **26** with hydrochloric acid in a mixture of methanol and THF after deprotection of the ketal unit [41] afforded a ring-opened product **27** in 39% yield with recovery of epoxy ketone **11** (42%). The structure of **27** was determined to be the 5-hydroxylated compound, not 8-oxygenated isomer **28**, by HMBC and NOE experiments (Figure 3).

**Table 2:** Effect of base on DAR of *in situ *formed benzyne **10** and furan (**9**).

Entry	Base (equiv)	Furan (equiv)	Conditions	**26** (%)^a^	**25** Recovery (%)^a^

1^b^	NaNH_2_ (4.0)	16	50 °C, 14 h	3	79
2^c^	NaNH_2_ (4.0)	16	100 °C, 250 W, 150 psi, 1 h	12	69
3	LDA (1.0)	14	−78 °C – rt, 3 h	25	53
4	LDA (2.0)	14	−78 °C – rt, 3.5 h	24	27
5	LDA (1.0)	2	−78 °C – rt, 3.5 h	30	39
6	(CH_3_)_2_Zn(TMP)Li (2.2)	2	-78 °C – rt, 3.5 h, 50 °C, 3 h	NR^d^	
7	LiTMP (1.0)	2	−78 °C – rt, 2.5 h	NR^d^	

^a^Isolated yield. ^b^Performed in a sealed tube. ^c^Under microwave irradiation. ^d^No reaction.

**Figure 4:**
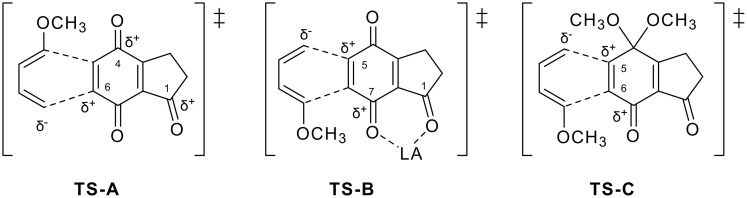
Transition states supposed for the regioselective DAR via quinone route.

**Figure 5:**
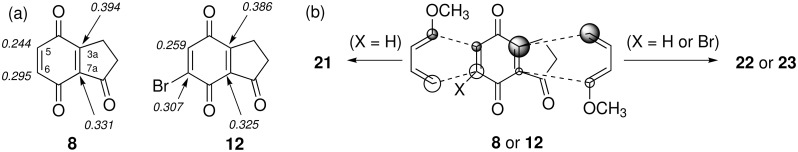
Representative LUMO coeffients of quinones **8** and **12** (a) and their reaction courses with diene **7** (b).

In the former quinone route, the regioselectivity on the introduced methoxy group in 2,3-dihydrobenz[*f*]indenone was examined. 4,5,9-Trioxygenated 2,3-dihydrobenz[*f*]indenone derivative **21** was exclusively formed on DAR of indanetrione **8** and diene **7** because of selective activation of the C6 carbon due to the presence of additional cross conjugation between the C1 and the C4 carbonyl groups (TS-A, Figure 4). Semiempirical calculation [44] of molecular orbitals of quinone **8** supported this proposal, in which a larger LUMO coefficient (0.295) was obtained at C6 compared with C5 (0.244, Figure 5a). On the other hand, the reverse of the selectivity was expected when Lewis acid is coordinated with two carbonyls at C1 and C7 to positively activate the C5 carbon (TS-B, Figure 4); however, the addition of a catalytic amount of Lewis acid did not affect the regioselectivity. Similar reversal of the selectivity was also expected on using a quinone monoacetal to mask the ketone functionality at the 4 position (TS-C); however, the desired 2,3-dihydrobenz[*f*]indenone-type compound was not obtained.

On the other hand, propellane-type product **22** was obtained as a by-product in DAR of **8**. In the case of reaction of bromoquinone **12**, propellane **23** was the sole product. Larger coefficients at C3a and C7a carbons compared with those of C5 and C6 ones supported this phenomenon (Figure 5a). The steric bulk of the bromine atom in **12** can assist the selective formation of **23** (Figure 5b).

**Scheme 4:**
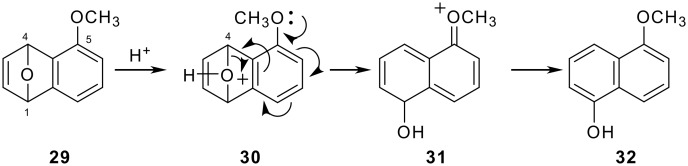
The proposed mechanism for the acid-induced ring opening of epoxynaphthalene **29** by Giles *et al. *[19].

**Scheme 5:**
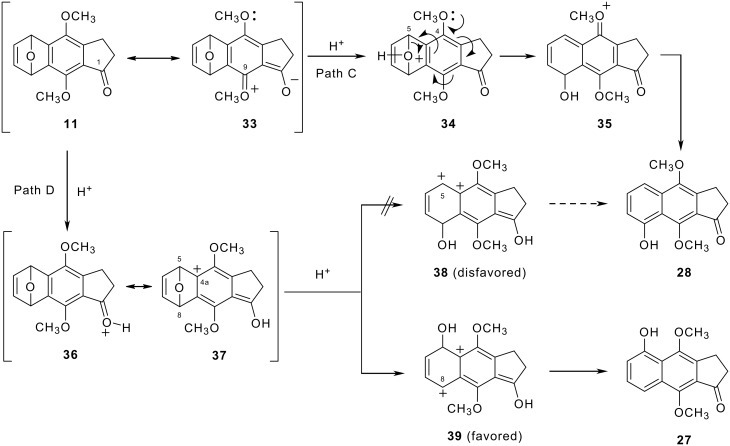
Supposed reaction pathways for the acid-induced ring opening of **11**.

In the latter benzyne strategy, 4,5,9-trioxygenated derivative **27** was formed as a sole product in the acid-catalyzed ring-opening of 5,8-epoxyindanone **11**. Giles *et al. *[41] reported the acid-induced ring opening of 1,4-epoxy-5-methoxynaphthalene (**29**) to furnish 5-methoxy-1-naphthol (**32**) via selective C4-O bond cleavage due to the electron-donating effect of the 5-methoxy group (Scheme 4). Therefore, in 5,8-epoxy-2,3-dihydrobenz[*f*]indenone system **11**, regioselective C5-O bond cleavage was expected by the aid of the 4-methoxy group due to the deactivation of the 9-methoxy group by conjugation with the carbonyl at the 1 position to afford desired 4,8,9-trioxygenated compound **28** (Path C, Scheme 5). However, protonation of the carbonyl oxygen of **11** could yield a C-4a carbocation **37** with conjugation to oxocarbenium ion **36** (path D). In this case, generation of the desired 4,8,9-trioxygenated 2,3-dihydrobenz[*f*]indenone **28** seemed unlikely due to unfavorable adjacent dicationic intermediate **38** after C5-O bond cleavage of **37**. Thus, formation of an alternative dication **39** through C8-O bond cleavage of **37** is favored instead to give undesired 4,5,9-trioxygenated 2,3-dihydrobenz[*f*]indenone **27**.

## Conclusion

DAR approaches toward regioselective construction of 4,8,9-trioxygenated 2,3-dihydrobenz[*f*]indenone skeleton were examined. Unfortunately undesired 4,5,9-trioxygenated derivatives were obtained; however, this finding could be applied to the synthesis of regioisomeric kinamycin analogues in each experiment. In the quinone route, the DAR has occurred mainly at the ring juncture of indanetriones to furnish propellane-type compounds [45]. This interesting framework of the products would be applicable to not only synthesis of other natural product but also preparation of newly designed functional molecules.

## Supporting Information

File 1 Experimental part
