# Supplementary material for: Diels-Alder reactions using 4,7-dioxygenated indanones as dienophiles for regioselective construction of oxygenated 2,3-dihydrobenz[f]indenone skeleton
Source: Beilstein J Org Chem. 2008 May 15;4:15. doi: 10.3762/bjoc.4.15 (PMC2486486; doi:10.3762/bjoc.4.15)
Supplement: File 1 — Experimental part [file Beilstein_J_Org_Chem-04-15-s001.doc]

**Supporting Information: Experimental.doc**

**Diels-Alder reactions using 4,7-dioxygenated indanones as dienophiles for regioselective construction of oxygenated 2,3-dihydrobenz[*f*]indenone skeleton**

Natsuno Etomi, Takuya Kumamoto*, Waka Nakanishi and Tsutomu Ishikawa*

Graduate School of Pharmaceutical Sciences, Chiba University

1-33 Yayoi-cho Inage-ku, Chiba 263-8522 Japan

Fax: +81-43-290-2911

* Corresponding author

Experimental part

All melting points were measured on a Yanagimoto MPSI melting point apparatus and are uncorrected. IR spectra were recorded as a Nujol mull or with attenuated total reflectance system (ATR) on a JASCO FT / IR-300E spectrophotometer. ^1^H and ^13^C NMR spectra were recorded in CDCl_3_ on a JEOL JNM-GSX-400α , JNM-ECP-400, or JNM-ECP-600. MS spectra were measured on a JEOL JNM MS-GC MATE for EIMS, and on a JEOL JMS-HX110 or a JEOL JMS-AX505 for FABMS. For column chromatography Kanto Chemical silica gel (SiO_2_) 60 spherical for normal, 60N spherical for neutral and Merck aluminium oxide (Al_2_O_3_) 90 (Brockman) were used. For preparative TLC (p-TLC) Merck Art 5744 DC-Fertigplatten Kieselgel 60 F_254_ was used. For TLC Merck Art 5715 DC-Fertigplatten Kieselgel 60 F_254_ was used. Anhydrous CH_2_Cl_2_ and THF were purchased from Kanto Chemical and Wako Chemical, respectively. Anhydrous CHCl_3_ was distilled from P_2_O_5_ before use. Microwave irradiation was done in a CEM Discover system in sealed tubes. Crude solutions after work-up were dried over Na_2_SO_4_ or MgSO_4_.

**4,7-Dimethoxyindan-1-one (17)**

CH_3_SO_3_H (5.4 mL, 83.1 mmol) was added to P_2_O_5_ (1.05 g, 7.40 mmol) under Ar atmosphere and the reaction mixture was stirred at 50 °C for 1 h. After cooling to rt, the reaction mixture was diluted with CH_2_Cl_2_ (10 mL) and a solution of acid **15** (434 mg, 2.06 mmol) in CH_2_Cl_2_ (8 mL) was added at rt. After stirring at rt for 19 h, the mixture was poured onto ice (50 mL), basified with 10% NaOH to pH 10 and extracted with CH_2_Cl_2_ (3 x 50 mL). The organic layers were combined and washed with sat. aq. NaHCO_3_ (1 x 70 mL), H_2_O (1 x 70 mL) and brine (1 x 70 mL), successively, then dried. The solvent was evaporated *in vacuo* and the residue was purified by column chromatography (CC) (Al_2_O_3_, *n*-hexane : AcOEt = 1 : 1) to give **17** as a pale yellow solid (266 mg, 67%).

mp 123-125 °C (lit. [1] 124-125 °C).

IR (Nujol): 1697 cm^-1^.

^1^H NMR (400 MHz): δ = 2.67 (t, *J=*5.9 Hz, 2H), 2.99 (t, *J=*5.9 Hz, 2H), 3.86 (s, 3H), 3.90 (s, 3H), 6.73 (d, *J=*8.7 Hz, 1H), 6.99 (d, *J=*8.7 Hz, 1H).

**6-Bromo-4,7-dimethoxyindan-1-one (18)**

Following the procedure for the synthesis of **17**, **18** (627 mg, 71%) was obtained as yellow solids from **16** (943 mg, 3.26 mmol), CH_3_SO_3_H (8.4 mL, 129 mmol) and P_2_O_5_ (1.66 g, 11.7 mmol) in CHCl_3_ (44 mL).

mp 110-112 °C (lit. [1] 109-110 °C).

Anal Calcd for C_11_H_11_BrO_3_: C, 48.73; H, 4.09. Found: C, 48.69; H, 4.10.

IR (Nujol): 1718 cm^-1^ (lit. [1] 1700 cm^-1^).

^1^H NMR (400 MHz): δ = 2.69 (t, *J=*5.9 Hz, 2H), 2.94 (t, *J=*5.9 Hz, 2H), 3.87 (s, 3H), 3.91 (s, 3H), 7.17 (s, 1H).

^13^C NMR (100 MHz): δ = 22.1, 36.6, 56.0, 61.9, 116.0, 119.4, 130.8, 145.1, 148.1, 152.9, 203.5.

EIMS: *m/z* (%) = 272 [M(^81^Br)^+^, 80%], 270 [M(^79^Br)^+^, 84%], 243 (100%), 241 (95%).

**4-Bromo-2,5-dimethoxyhydrocinnamic acid (16)**

Bromine water (2.62 mmol as Br_2_), prepared by addition of 5% aq. NaOCl (3.8 g, 2.62 mmol) to an ice-cooled solution of NaBr (539 mg, 5.24 mmol) and 1N HCl (5.3 mL, 5.30 mmol) in H_2_O (17 mL), was added to a solution of **15** (501 mg, 2.38 mmol) in 1,4-dioxane - H_2_O (4 : 1, 2.4 mL) under ice-cooling within 75 min and the reaction mixture was stirred at rt for 2 h. The precipitates were filtered off, washed with H_2_O and dried under air to give colorless solids, which were recrystallized from EtOH to give **16** as colorless prisms (465 mg, 68%)

mp 134-137 °C (lit. [1] 136-137 °C).

IR (Nujol): 3300, 1699 cm^-1^.

^1^H NMR (400 MHz) δ = 2.65 (t, *J*=7.6 Hz, 2H), 2.90 (t, *J*=7.6 Hz, 2H), 3.78 (s, 3H), 3.84 (s, 3H), 6.78 (s, 1H), 7.02 (s, 1H).

**2,3-Dihydroindene-1,4,7-trione (8)**

To a solution of **17** (199 mg, 1.04 mmol) in acetonitrile (5 mL), a solution of CAN (1.43 g, 2.61 mmol) in H_2_O (15 mL) was added under ice-cooling within 10 min. The resultant orange solution was stirred at 0 °C for 30 min. The whole was extracted with CHCl_3_ (3 x 15 mL) and the combined organic layers were washed with H_2_O (2 x 10 mL) and brine (2 x 10 mL), successively, and dried. The solvent was evaporated *in vacuo* and the residue was washed with a mixture of AcOEt - Et_2_O (5 : 1) to give **8** as a brown solid (110 mg, 65%). This compound was used immediately to the next step because of its instability.

mp 78-90 °C.

IR (Nujol): 1718, 1655 cm^-1^.

^1^H NMR (400 MHz): δ = 2.65 (t, *J=*5.1 Hz, 2H), 2.94 (t, *J=*5.1 Hz, 2H), 6.81 (d, *J=*10.1 Hz, 1H), 6.90 (d, *J=*10.1 Hz, 1H).

^13^C NMR (100 MHz): δ = 22.7, 35.2, 135.7, 136.5, 137.3, 168.4, 182.4, 186.6, 203.2.

**7-Hydroxy-4-methoxyindan-1-one (19)**

A suspension of **17** (851 mg, 4.43 mmol) and MgI_2_ · 6 H_2_O (1.76 g, 4.56 mmol) in benzene (85 mL) was refluxed for 10 h in a Dean-Stark apparatus. After cooling to rt, H_2_O (40 mL) and 10% HCl (40 mL) were added and the reaction mixture was extracted with AcOEt (3 x 80 mL). The combined organic layers were washed with H_2_O (2 x 80 mL) and brine (1 x 80 mL) and dried. The solvent was evaporated *in vacuo* and the residue was recrystallized from EtOH to give **19** as pale orange prisms (636 mg, 81%).

mp 122-124 °C. (lit. [2] mp 121-122 °C)

IR (Nujol): 3422, 1685 cm^-1^.

^1^H NMR (400 MHz): δ = 2.71 (t, *J=*5.6 Hz, 2H), 3.04 (dif. t, *J=*5.6 Hz, 2H), 3.84 (s, 3H), 6.73 (d, *J=*8.7 Hz, 1H), 7.00 (d, *J=*8.7 Hz, 1H), 8.55 (s, 1H).

^13^C NMR (100 MHz): δ = 22.8, 35.9, 56.0, 113.8, 119.1, 123.4, 142.3, 149.7, 160.7, 210.1.

EIMS: *m/z* (%) = 178 (M^+^, 100).

**6-Bromo-7-hydroxy-4-methoxyindan-1-one (20)**

Following the procedure for the synthesis of **19**, **20** (461 mg, 96%) was obtained as a pale yellow solid from **18** (508 mg, 1.88 mmol), MgI_2_ · 6 H_2_O (728 mg, 1.88 mmol) and benzene (50 mL).

mp 146-147.5 °C.

Anal Calcd for C_10_H_9_BrO_3_: C, 46.72; H, 3.53. Found: C, 46.36; H, 3.53.

IR (Nujol): 3361, 1671 cm^-1^.

^1^H NMR (400 MHz): δ = 2.75 (t, *J=*5.5 Hz, 2H), 2.99 (t, *J=*5.5 Hz, 2H), 3.84 (s, 3H), 7.18 (s, 1H), 9.00 (s, 1H).

^13^C NMR (100 MHz): δ = 22.6, 35.9, 56.2, 105.8, 121.6, 123.7, 142.2, 147.4, 149.9, 209.3.

EIMS: *m/z* (%) = 258 [M(^81^Br)^+^, 90%], 256 [M(^79^Br)^+^, 100%].

**6-Bromo-2,3-dihydroindene-1,4,7-trione (12)**

To a suspension of **20** (58 mg, 0.22 mmol) in acetonitrile - H_2_O (2 : 1, 0.7 mL), a solution of PIFA (192 mg, 0.45 mmol) in acetonitrile - H_2_O (2 : 1, 1.4 mL) was added at rt and the reaction mixture was stirred at rt for 30 min. H_2_O (2 mL) was added and the reaction mixture was extracted with CH_2_Cl_2_ (2 x 5 mL). The combined organic layers were washed with H_2_O (1 x 5 mL) and brine (1 x 5 mL) successively, and dried. The solvent was evaporated *in vacuo* and the residue was washed with Et_2_O to give **12** as an orange solid (47 mg, 86%). This compound was used immediately to the next step because of its instability.

mp 128 °C (dec.)

IR (Nujol):1720, 1670 cm^-1^.

^1^H NMR (400 MHz): δ = 2.70 (t, *J=*5.1 Hz, 2H), 2.95 (t, *J=*5.1 Hz, 2H), 7.43 (s, 1H).

^13^C NMR (100 MHz): δ = 23.0, 35.5, 136.3, 138.3, 140.4, 169.1, 174.3, 183.6, 201.5.

**4,4-Dimethoxy-2,3-dihydro-4*H*-indene-1,7-dione (13)**

Following the procedure for the synthesis of **12**, **13** (175 mg, 74%) was obtained as a pale yellow solid from **19** (202 mg, 1.13 mmol) in methanol (4.5 mL) and PIFA (499 mg, 1.13 mmol) in acetonitrile (2.3 mL)

mp 81-85 °C.

IR (Nujol): 1725, 1675 cm^-1^.

^1^H NMR (400 MHz): δ = 2.59 (t, *J=*5.1 Hz, 2H), 2.85 (t, *J=*5.1 Hz, 2H), 3.36 (s, 6H), 6.44 (d, *J=*10.4 Hz, 1H), 6.91 (d, *J=*10.4 Hz, 1H).

^13^C NMR (100 MHz): δ = 24.1, 35.0, 51.3, 94.2, 133.1, 135.0, 141.8, 180.1, 184.3, 202.5.

HREIMS: *m/z* [M^+^] Calcd for C_11_H_12_O_4_: 208.0735; found: 208.0720.

**6-Bromo-4,4-dimethoxy-2,3-dihydro-4*H*-indene-1,7-dione (14)**

Following the procedure for the synthesis of **13**, **14** (63 mg, 92%) was obtained as a pale yellow solid from **20** (61 mg, 0.28 mmol) in methanol (1.0 mL) and PIFA (103 mg, 0.24 mmol) in acetonitrile (0.5 mL). An aliquot was recrystallized from methanol to give pale yellow needles.

mp 135-137 °C (dec).

IR (Nujol): 1734 cm^-1^.

^1^H NMR (400 MHz): δ = 2.61 (t, *J=*5.1 Hz, 2H), 2.85 (t, *J=*5.1 Hz, 2H), 3.38 (s, 6H), 7.38 (s, 1H).

^13^C NMR (100 MHz): δ = 24.0, 35.0, 51.5, 95.2, 128.9, 133.8, 142.4, 172.6, 184.3, 201.2.

HREIMS: *m/z* [M^+^] Calcd for C_11_H_11_^79^BrO_4_: 285.9840; found: 285.9843.

**DAR of indanetrione 8 and diene 7** (entry 1 in **Table 1**)

To a solution of **8** (160 mg, 0.99 mmol) in CH_2_Cl_2_ (1.6 mL), **7** (224 mg, 2.61 mmol) in CH_2_Cl_2_ (1.6 mL) was added at -16 °C and the reaction mixture was stirred at -16 °C for 3 h. The solvent was evaporated *in vacuo* and the residue was purified by column chromatography (SiO_2_, hexane - AcOEt = 2 : 1 to 1 : 1) to give a less polar propellane **22** as yellow needles (101 mg, 42%) and a more polar 2,3-dihydrobenz[*f*]indenone **21** as yellow needles (113 mg, 47%).

**(4a*SR*,8a*SR*)-8-Methoxy-5,8-dihydro-4a,8a-propanonaphthalene-1,4,9-trione (22)**

mp 100-103 °C (dec), 125-127 °C (melt).

Anal Calcd for C_14_H_14_O_4_: C, 68.28; H, 5.73. Found: C, 68.22; H, 5.80.

IR (Nujol): 1751, 1683, 1672 cm^-1^.

^1^H NMR (400 MHz): δ  = 1.70 (dddd, *J=*19.3, 4.6, 2.6, 1.9 Hz, 1H), 1.98 (m, 1H), 2.27 (m, 1H), 2.32 (m, 1H), 2.56 (m, 1H), 3.22 (s, 3H), 3.33 (ddd, *J=*19.3, 4.9, 2.0 Hz, 1H), 4.22 (dd, *J=*4.1, 1.7 Hz, 1H), 5.87 (ddd, *J=*10.2, 4.6, 2.7 Hz, 1H), 5.94 (dddd, *J=*10.5, 4.1, 2.2, 1.9 Hz, 1H), 6.70 (d, *J=*1.7 Hz, 2H).

^13^C NMR (150 MHz): δ = 26.8, 33.3, 34.1, 50.1, 57.6, 71.3, 74.6, 123.9, 129.1, 137.8, 139.6, 193.1, 198.2, 206.3.

EIMS: *m/z* (%) = 246 (M^+^, 0.02%), 190 (42%), 84 (100%).

**(4a*SR*,5*RS*,8a*SR*)-5-Methoxy-2,3,4a,5,8,8a-hexahydro-1*H*-benz[*f*]inden-1,4,9-trione (21)**

mp 130-142 °C (dec), 158 °C (melt).

IR (Nujol): 1757, 1681 cm^-1^.

^1^H NMR (600 MHz): δ = 2.09 (dd, *J=*20.2, 7.5 Hz, 1H), 2.61-2.64 (m, 2H), 2.80-2.83 (m, 2H), 3.11 (s, 3H), 3.14 (d, *J=*20.2 Hz, 1H), 3.25 (dd, *J=*6.8, 6.8 Hz, 1H), 3.30 (dd, *J=*6.8, 4.2 Hz, 1H), 3.99 (1H, dd, *J=*4.1, 2.8 Hz, 1H), 6.00 (m, 2H).

^13^C NMR (150 MHz): δ = 21.4, 22.7, 35.8, 44.3, 52.9, 56.1, 72.6, 123.3, 131.1, 142.2, 172.1, 191.1, 199.8, 204.0.

HRFABMS: *m/z* [M+H^+^] Calcd for C_14_H_15_O_4_: 247.0970, found: 247.0973.

**DAR of bromoindanetrione 12 and diene 7: (4a*SR*,8a*SR*)-2-Bromo-8-methoxy-5,8-dihydro- 4a,8a-propanonaphthalene-1,4,9-trione (23)** (entry 6 in **Table 1**)

Following the procedure for the synthesis of **21** and **22**, **23** (121 mg, 89%) was obtained as a yellow oil from **12** (101 mg, 0.42 mmol) in CH_2_Cl_2_ (4.5 mL), **7** (93 mg, 1.11 mmol) and CH_2_Cl_2_ (5.5 mL). An aliquot was solidified with keeping in refrigerator for two weeks and washed with Et_2_O to give a pale yellow powder.

mp 109-111.5 °C.

IR (Nujol): 1757, 1655 cm^-1^.

^1^H NMR (400 MHz): δ = 1.67 (ddd, *J*=18.8, 4.6, 2.4 Hz, 1H), 2.00 (ddd, *J*=13.8, 9.3, 2.4 Hz, 1H), 2.23 (m, 1H), 2.33 (m, 1H), 2.56 (ddd, *J*=19.2, 9.5, 1.8 Hz, 1H), 3.25 (s, 3H), 3.29 (ddd, *J*=18.8, 4.9, 1.6 Hz, 1H), 4.24 (br. d, *J*=2.0 Hz, 1H), 5.85 (ddd, *J*=10.2, 4.9, 2.4 Hz, 1H), 5.92 (1H, dddd, *J*=10.2, 4.0, 2.2, 1.7 Hz, 1H), 7.21 (s, 1H).

^13^C NMR (150 MHz): δ = 26.6, 32.4, 33.5, 51.0, 57.9, 70.4, 74.3, 123.7, 128.5, 139.2, 140.5, 186.4, 194.7, 205.1.

HRFABMS: *m/z* [M+H^+^] Calcd for C_14_H_14_^79^BrO_4_: 325.0075: found: 325.0080.

**DAR of ketal 13 and diene 7 under thermal conditions** (entry 9 in **Table 1**)

A solution of **13** (33 mg, 0.16 mmol) and **7** (36 mg, 0.43 mmol) in toluene (0.6 mL) was refluxed in a sealed tube at 120 °C (bath temp) for 9 h. After cooling to rt, the solvent was evaporated *in vacuo* and the residue was purified by column chromatography (SiO_2_, benzene - AcOEt = 1 : 0 to 4 : 1) to give **19** (6 mg, 21%) and **22** (6 mg, 16%).

**DAR of bromoketal 14 and diene 7 under Lewis-acid conditions** (entry 11 in **Table 1**)

To a mixture of ZnCl_2_ (activated by heating with a heat gun under vacuum, 10 mg, 0.08 mmol) and **14** (103 mg, 0.36 mmol) in CH_2_Cl_2_ (0.9 mL), a solution of **7** (84 mg, 1.00 mmol) in CH_2_Cl_2_ (0.8 mL) was added at -18 °C and the whole was stirred at rt for 2 h. H_2_O (1.5 mL) was added and the reaction mixture was extracted with CH_2_Cl_2_ (2 x 5 mL). The combined organic layers were washed with H_2_O (1 x 5 mL) and brine (1 x 5 mL), then dried. The solvent was evaporated *in vacuo* and the residue was purified by CC (SiO_2_, hexane - AcOEt = 4 : 1) to give **20** (14 mg, 15%) and **23** (9 mg, 8%).

**6-Bromo-1,1-ethylenedioxy-4,7-dimethoxyindane (25)**

A mixture of **18** (1.25 g, 4.61 mmol), PPTS (1.16 g, 4.63 mmol) and ethylene glycol (2.60 mL, 46.6 mmol) in benzene (16 mL) was refluxed in a Dean-Stark apparatus for 17 h. The mixture was cooled to rt and the reaction was quenched by addition of H_2_O (20 mL) and sat. aq. NaHCO_3_ (20 mL). The aqueous layer was separated and extracted with AcOEt (3 x 20 mL). The combined organic layers were washed with sat. aq. NaHCO_3_ (1 x 20 mL), H_2_O (1 x 20 mL), and brine (1 x 20 mL), then dried. The solvent was evaporated *in vacuo* and the residue was purified by CC (SiO_2_, hexane - AcOEt = 10 : 1) to give **25** as a colorless powder (1.28 g, 89%).

mp 66-71 °C

IR (ATR) no characteristic absorption.

^1^H NMR (400 MHz): δ = 2.28 (t, *J*=6.9 Hz, 2H), 2.77 (t, *J*=6.9 Hz, 2H), 3.79 (s, 3H), 3.85 (s, 3H), 4.03-4.09 (m, 2H), 4.18-4.24 (m, 2H), 6.95 (s, 1H).

^13^C NMR (100 MHz): δ = 24.6, 37.8, 55.7, 61.5, 65.6, 115.4, 115.6, 117.8, 133.4, 136.8, 147.1, 152.5.

HRFABMS: *m/z* [M+H^+^] Calcd for C_13_H_16_^79^BrO_4_: 314.0232; found 315.0244.

**DAR of the ketal 25 and furan (9): 5,8-epoxy-1,1-ethylenedioxy-4,9-dimethoxy-2,3,5,8-tetrahydro- 1*H*-benz[*f*]indene (26) (i) with NaNH_2_** (entry 1 in **Table 2**) To a mixture of NaNH_2_ (60 mg, 1.54 mmol) in THF (0.15 mL) was added a solution of **9** (0.45 mL, 6.20 mmol) and **25** (121 mg, 0.39 mmol) in THF (0.55 mL) and the reaction mixture was stirred in a sealed tube at 50 °C for 14 h. After cooling to rt, H_2_O (4 mL) was added and the whole was extracted with AcOEt (3 x 5 ml). The combined organic layers were washed with H_2_O (1 x 5 mL) and brine (1 x 5 mL) and dried. The solvent was evaporated *in vacuo* and the residue was purified by CC (neutral SiO_2_, *n*-hexane : AcOEt = 5 : 1 to 2 : 1) to give **26** as a pale yellow oil (4 mg, 3%) and also some recovered **25** (97 mg, 79%). **26** solidified on standing and was washed with Et_2_O to give a beige solid.

mp 129-132 ºC.

IR (ATR)  no characteristic absorption.

^1^H NMR (400 MHz): δ = 2.29 (t, *J*=7.0 Hz, 2H), 2.76 (t, *J*=7.0 Hz, 2H), 3.85 (s, 3H), 3.87 (s, 3H), 3.98-4.03 (m, 2H), 4.13-4.20 (m, 2H), 5.97 (brs, 1H), 5.99 (brs, 1H), 7.00 (dd, *J*=5.3, 1.6 Hz, 1H), 7.02 (dd, *J*=5.3, 1.6 Hz, 1H).

^13^C NMR (100 MHz): δ = 24.8, 38.5, 60.17, 60.18, 65.6, 65.6, 80.72, 80.73, 117.7, 132.2, 136.1, 137.3, 138.4, 142.1, 142.5, 145.4, 145.7.

HREIMS: *m/z* [M^+^] Calcd for C_17_H_18_O_5_: 302.1154; found: 302.1136.

**(ii) With LDA** (entry 5 in **Table 2**)

To a solution of diisopropylamine (0.05 mL, 0.38 mmol) in THF (0.2 mL), *n*-BuLi (1.45 M, 0.22 mL, 0.32 mmol) was added at -78 °C and the reaction mixture was stirred at 0 °C for 15 min. After cooling at -78 °C, a solution of **9** (0.05 mL, 0.69 mmol) and **25** (101 mg, 0.32 mmol) in THF (0.4 mL) was added and the reaction mixture was warmed gradually to rt within 3 h and stirred at the same temperature for 1 h. The mixture was diluted with AcOEt (3 mL) and washed with H_2_O (1 x 2 mL) and brine (1 x 2 mL), then dried. The solvent was evaporated *in vacuo* and the residue was purified by column chromatography (neutral SiO_2_, *n*-hexane: AcOEt = 5 : 1 to 2 : 1) to give **26** (29 mg, 30%) with 39% recovery of **25** (39 mg,).

**5,8-Epoxy-4,9-dimethoxy-2,3,5,8-tetrahydrobenz[*f*]inden-1-one (11)**

An aliquot (1.0 mL) of a solution of *conc.* HCl in methanol (1 drop / 8 mL) was added to **26** (45 mg, 0.15 mmol) and the reaction mixture was refluxed for 19 h. After cooling, the solvent was evaporated *in vacuo* and the residue was partitioned with AcOEt (10 mL) and H_2_O (5 mL). The organic layer was washed with H_2_O (1 x 5 mL) and brine (1 x 5 mL), then dried. The solvent was evaporated *in vacuo* and the residue was washed with Et_2_O followed by recrystallization from EtOH to give **11** as a gray solid (19 mg, 50%).

mp 149-151 °C.

IR (ATR): 1697 cm^-1^.

^1^H NMR (400 MHz) δ = 2.66-2.69 (m, 2H), 2.93 (t, *J*=5.7 Hz, 2H), 3.94 (s, 3H), 3.97 (s, 3H), 6.04 (dd, *J*=1.8, 0.9 Hz, 1H), 6.06 (dd, *J*=1.8, 0.9 Hz, 1H), 7.02 (dd, *J*=5.6, 1.8 Hz, 1H), 7.08 (dd, *J*=5.6, 1.8 Hz, 1H).

^13^C NMR (100 MHz) δ = 22.5, 37.4, 60.2, 61.3, 80.4, 80.9, 128.3, 137.2, 141.1, 143.2, 143.7, 145.9, 146.1, 150.2, 203.9.

HREIMS: *m/z* [M^+^] Calcd for C_15_H_14_O_4_: 258.0892; found: 258.0873.

**5-Hydroxy-4,9-dimethoxy-2,3-dihydrobenz[*f*]inden-1-one (27)**

An aliquot (0.4 mL) of a solution of *conc.* HCl in methanol (1 drop / 1 mL) and THF (0.2 mL) was added to **11** (14 mg, 0.053 mmol) and the reaction mixture was refluxed for 38 h. After cooling, the solvent was evaporated *in vacuo* and the residue was partitioned with AcOEt (5 mL) and H_2_O (3 mL). The organic layer was washed with H_2_O (1 x 5 mL) and brine (1 x 5 mL), then dried. The solvent was evaporated *in vacuo* and the residue was purified by preparative TLC (*n*-hexane - AcOEt = 3 : 1 x 5) to give **27** as a yellow solid (5 mg, 39%) with 42% recovery of **11** (6 mg).

mp 157-158 °C.

IR (ATR): 3302, 1705 cm^-1^.

^1^H NMR (400 MHz) δ = 2.80 (diffused t, *J*=6.9 Hz, 2H), 3.29 (diffused t, *J*=6.9 Hz, 2H), 4.07 (s, 3H), 4.15 (s, 3H), 7.05 (dd, *J=*7.5, 0.9 Hz, 1H), 7.41 (dd, *J*=8.4, 7.5 Hz, 1H), 7.88 (dd, *J*=8.4, 0.9 Hz, 1H), 9.57 (s, 1H).

^13^C NMR (100 MHz) δ = 22.3, 37.3, 61.8, 63.2, 113.8, 115.9, 121.3, 123.9, 127.4, 130.6, 135.7, 147.6, 152.6, 153.7, 203.5.

HREIMS: *m/z* [M^+^] Calcd for C_15_H_14_O_4_: 258.0892; found: 258.0899.

References

1. Coutts, R. T.; Malicky, J. L. *Can. J. Chem.* **1974,** *52,* 381–389. doi:[10.1139/v74-061](http://dx.doi.org/10.1139/v74-061)
2. Horaguchi, T.; Tamura, S.; Hiratsuka, N.; Suzuki, T. *J. Chem. Soc., Perkin Trans. 1* **1985,** 1001–1006. doi:[10.1039/P19850001001](http://dx.doi.org/10.1039/P19850001001)
